# Statistical quality assessment and outlier detection for liquid chromatography-mass spectrometry experiments

**DOI:** 10.1186/1756-0381-2-4

**Published:** 2009-04-07

**Authors:** Ole Schulz-Trieglaff, Egidijus Machtejevas, Knut Reinert, Hartmut Schlüter, Joachim Thiemann, Klaus Unger

**Affiliations:** 1 International Max Planck Research School for Computational Biology and Scientific Computing, Berlin, Germany; 2 Department Computer Science and Mathematics, Freie Universität Berlin, Berlin, Germany; 3 Institute for Anorganic and Analytical Chemistry, Johannes Gutenberg-Universität, Mainz, Germany; 4 Core Facility Protein Analytics, Charité – Universitätsmedizin Berlin, Berlin, Germany

## Abstract

**Background:**

Quality assessment methods, that are common place in engineering and industrial production, are not widely spread in large-scale proteomics experiments. But modern technologies such as Multi-Dimensional Liquid Chromatography coupled to Mass Spectrometry (LC-MS) produce large quantities of proteomic data. These data are prone to measurement errors and reproducibility problems such that an automatic quality assessment and control become increasingly important.

**Results:**

We propose a methodology to assess the quality and reproducibility of data generated in quantitative LC-MS experiments. We introduce quality descriptors that capture different aspects of the quality and reproducibility of LC-MS data sets. Our method is based on the Mahalanobis distance and a robust Principal Component Analysis.

**Conclusion:**

We evaluate our approach on several data sets of different complexities and show that we are able to precisely detect LC-MS runs of poor signal quality in large-scale studies.

## Background

**Figure 1:**
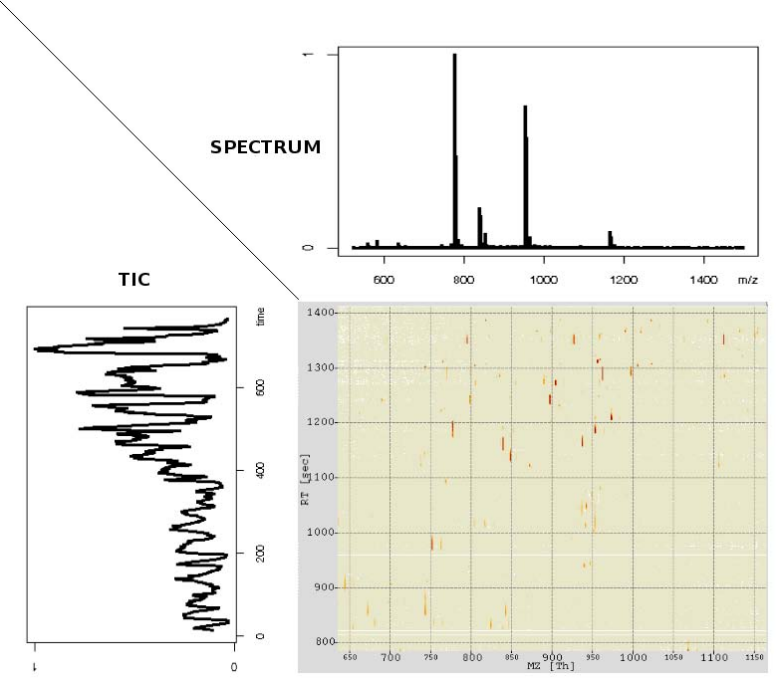
**An LC-MS map obtained from human serum**. An LC-MS map obtained from human serum [5]. It consists of a sequence of spectra, such as the one shown on top. The projection on RT, the Total Ion Chromatogram (TIC), is shown on the left.

Mass spectrometry has become a cornerstone of research in proteomics [1]. Especially the combination of liquid chromatography and mass spectrometry (LC-MS) has shown great promise for basic research and clinical studies. In this setup, a chromatographic column is coupled to the mass spectrometer. The chromatography leads to a first separation and simplification of the sample. The mass spectrometer records a set of mass spectra for the sample and each spectrum contains the signals for a subset of the sample compounds eluting from the column at a specific retention time (rt). We will call the outcome of an LC-MS experiment the LC-MS map. This term denotes the collection of all mass spectra that have been obtained from this LC-MS run [2-4]. The LC-MS map is a set of points in 3D, each point characterized by m/z, rt and intensity. Since the m/z dimension is relatively stable across replicates, a helpful diagnostic plot is the projection of the LC-MS map on the retention time. This plot is called the Total Ion Chromatogram (TIC) as shown in Figure 1 for a map obtained from human serum [5].

This work addresses the problem of quality assessment in large scale LC-MS studies. So far, this is a relatively unexplored topic. There are some publications on the quality assessment of MS fragmentation spectra [6-12]. But their focus is different: the aim of these methods is to detect and remove low quality spectra from a LC-MS/MS run. The rationale is that these spectra would not be identified by identification algorithms anyway and that their removal will lead to a significant speed-up of the data analysis. To give some examples, Bern *et al*. [6] pioneered the quality filtering of MS/MS spectra. They used various descriptors to describe an MS/MS such as the number of isotopic peaks or the number of peaks that could be clearly attributed to *b *or *y *fragments. Bern *et al*. applied support vector machines and linear discriminants and removed MS/MS spectra classified as poor before the database search. Since then, several works tried to improve on this approach. Notable developments include the application of self-convolution for MS/MS quality assessment [7] or an iterative strategy to detect high-quality spectra that could not be identified in the database search [9].

Our work, however, addresses a different problem. In a quantitative LC-MS experiment, the aim is to obtain abundance estimates of all peptides and proteins contained in a sample. Fragmentation and sequencing of the peptides using MS/MS is usually an additional experimental step and is not the focus of this work. Controlling data quality in high-throughput experiments as early possible is important since numerous problems can affect the quality of an LC-MS run. Among these are instabilities of the chromatography, degradation of the peptides or artifacts in the mass spectra caused by the LC mobile phase or buffer molecules.

Only little work on the quality and reproducibility assessment of mass spectrometry data has been published so far [13-16]. Prakash et al. [15] use a distance measure computed by an alignment algorithm to highlight problems of reproducibility in several mass spectrometry studies. Their method is successful in visualizing the time order in which the LC-MS runs were performed and reveals pattern caused by different columns or instrument settings used. But their method does not provide direct information on outlier runs and when to discard them. Whistler *et al*. [16], Coombes *et al*. [13] and Harezlak *et al*. [14] address the problem of noise removal and quality assessment but focus solely on SELDI-TOF spectra which are less complex than LC-MS data.

The analysis of LC-MS data is a sophisticated task and requires several computational steps such as denoising, peptide feature detection, alignment and statistical analysis [17]. After differentially expressed peptide feature have been found, they need to be sequenced using MS/MS-based identification and have their abundances and sequences mapped to the parent protein. These are general steps which usually have to be adopted depending on the aim of the study. But each of these computational steps has its own difficulties and a typical workflow is complex and error prone [18]. It is therefore desirable to identify poor LC-MS runs as early as possible. This would allow us to either exclude these runs from the further analysis, to repeat them or at least to downweight these measurements to reflect our reduced confidence. In contrast to mass spectrometry-based proteomics, quality assessment and control methods are more common in gene expression studies [19-21]. Brown *et al*. [19] applied image metrics to find poor quality microarrays in a batch of experiments. Cohen *et al*. [20] applied the Mahalanobis distance to detect outlier runs in large-scale gene expression studies. Finally, Model *et al*. [21] borrowed methods from the field of statistical process control to detect critical differences among replicate microarray measurements.

In this work, we investigate how classical methods from outlier detection and quality control can be extended and applied to LC-MS data. Our approach is based on sound statistical principles and we demonstrate that we can precisely detect dubious LC-MS runs in large scale studies.

## Results

This work addresses the quality assessment of raw LC-MS maps. By "raw", we mean the unprocessed spectra before any noise filtering, peak detection or centroiding has been performed. Most statistical methods for quality assessment expect that each item is described by one (univariate) or several (multivariate) variables. For LC-MS maps, it is not clear what suitable variables could be. One straightforward approach is to describe an LC-MS map by all its data points. But the number of data points (not peaks) in an unprocessed LC-MS map is huge, easily several millions of points. Second, many of the raw data points in a map will be caused by noise and might distort the results of an automatic outlier detection.

Consequently, we devised a list of quality descriptors to describe an LC-MS map. Some of these descriptors were taken from the literature, where they have been shown to be useful criteria for spectra mining and filtering tasks, others are new. Using these quality descriptors, we can now describe each map as a vector  = (*x*_1_, *x*_2_,...,*x*_*n*_)^*T *^and apply statistical methods to detect runs of poor quality.

We emphasize that we define quality in terms of reproducibility, i.e., an LC-MS map is of poor quality if its quality descriptors differ significantly from the descriptors of the other maps. It is thus important to compare only maps that represent the same subsets of a sample. As an example, in a multidimensional chromatography experiment, we can only compare LC-MS recordings of the same chromatography steps. It does not make sense to compare LC-MS maps obtained from different salt pulses, to give an example. On the other hand, even for time series or differential quantitative measurements, we would still expect the key characteristics of the LC-MS maps, such as noise level or chromatography, to remain stable during the study.

### Algorithms

We use a set of quality descriptors to an LC-MS map. These descriptors capture various aspects of the map, such as peaks and noise level of the spectra, as well shape and reproducibility of the TIC. The descriptors are:

• Median of the Euclidean distances *D*_*E*_(*s*, *s'*) =  between baseline-removed spectrum *s' *and original spectrum *s *for all spectra. The baseline or background noise in a mass spectrum is usually caused by molecules from the mobile phase of the column. Spectra with a large amount of background noise are difficult to analyze automatically. This rationale of this descriptor is that spectra with a strong baseline signal will be very different after baseline removal and thus have a large distance *D*_*E*_. We perform the baseline removal using a TopHat-Filter which is a standard method for this task.

• Median of the Euclidean distances *D*_*E *_between smoothed mass spectrum and original spectrum for all spectra. Consequently, a noisy spectrum will exhibit a large distance *D*_*E *_to its smoothed version. We performed the smoothing using a Gaussian Filter with a kernel width of ≈2.0, depending on the mass spectral peak width. The first two quality descriptors were firstly suggested by Windig *et al*. [22] but they applied them to chromatograms to remove noisy mass traces from the LC-MS map.

• The *Xrea *value. This measure for the quality of a mass spectrum was already proposed by Na *et al*. [8]. They developed it to filter MS/MS spectra before submitting them to a sequence database search. We will show that the *Xrea *criterion can equally be applied to MS spectra. The *Xrea *value is based on a cumulative intensity normalization. First, we normalize the spectral intensities by dividing by the total intensity. The *cumulative normalized intensity *of each data point in the spectrum is defined as the sum of the normalized intensities of all points with intensities smaller than or equal to the intensity of this point. Accordingly, the cumulative normalized intensity of the *n*th highest data point *x *is given by:

(1)

where *I*(*x*) is the intensity of point *x *and Rank(*x*) represents the order of points if sorted by intensity in descending order. That is, the most intense point has rank 1, the next rank 2 etc. The nominator is divided by the sum of all intensities. This normalization is relatively stable and less dependent on the most intense peak which is a disadvantage of a normalization by the intensity sum. But in contrast to other methods such as a rank-based normalization, it does not discard the entire information contained in the spectral intensities.

**Figure 2:**
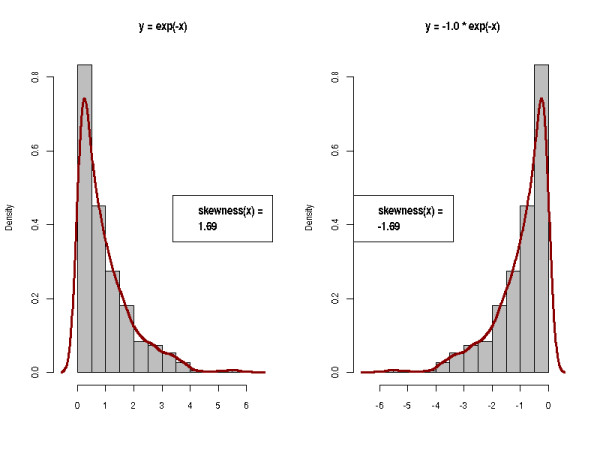
**The Xrea value**. Two examples of the Xrea value: spectrum A is of good quality with low background noise where spectrum B has a large amount of noise. The plots to the left show the corresponding Xrea distribution. The area denoted by *XX *is used in Equation 2.

We use the distribution of cumulative intensities to derive a measure for the quality of a spectrum. Figure 2 shows a plot of two spectra and their cumulative intensities. Spectrum A would usually be considered a good quality spectrum: several peaks with high intensity and only a low amount of background noise. Spectrum B is of poor quality: it contains a lot of noise signals and a strong background signal. To the left of each spectrum, we give the distribution of Xrea values. Its upper bound is the plot of each point versus its rank, indicated by the green line. We can see that, for spectra with a more uniform distribution, the distribution of cumulative intensities approximates the diagonal. For spectra with less background noise, but some pronounced peaks, the distribution is more peak-shaped. Note that the spectra to the right are MS and not MS/MS spectra.

Flikka *et al*. proposed to use the area between the diagonal and the cumulative intensity distribution as an indicator of the quality of a spectrum. In the plots in Figure 2 this area is indicated by *XX*. If this area is large, the spectrum has low background noise and some elevated peaks of high intensity, which is desirable for feature detection and quantification. If this area is small, all intensities in the spectrum are similar and the information content of this spectrum is rather low. To sum up, the Xrea quality descriptor is given by

(2)

where *α *is a correction term to account for cases in which the highest point is significantly larger than the rest. Following [12], we set *α *to the relative intensity of the most abundant data point.

**Figure 3:**
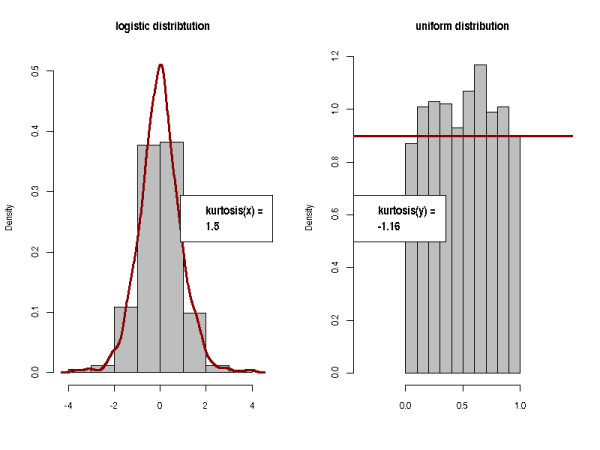
**Skewness of a distribution**. The skewness of a distribution is positive if the distribution is right-tailed. It is negative if the distribution has a tail to the left.

**Figure 4:**
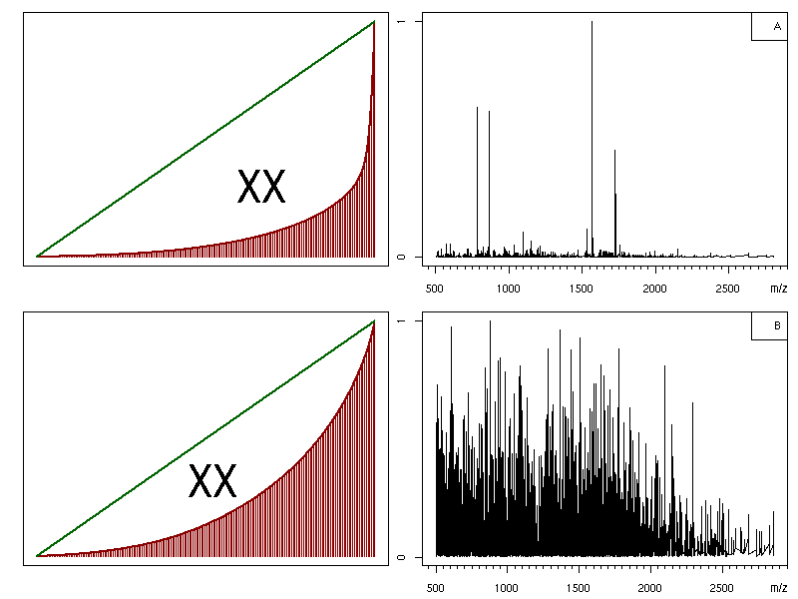
**Kurtosis of a distribution**. The Kurtosis measures whether a distribution is rather peak-shaped or flat relative to a Gaussian distribution.

• Median of the number of data points with intensity ≥ 0 in each scan. This descriptor accounts for variations in the number of recorded intensities.

• Summary statistics for m/z, intensity and signal-to-noise ratio of all scans. The summary statistics consist of minimum, maximum, mean and median. We estimate the noise level using an iterative sliding window approach. We move a window of size 25 Th across each spectrum and calculate a noise level for each window. We compute mean and standard deviation *σ *of all intensities in the current window and discard all points with an intensity higher than 3 × *σ*. We repeat this procedure and estimate the local noise level as the medium intensity after three iterations.

• Skewness and kurtosis of the TIC. For good and reproducible LC-MS runs, the TICs should exhibit similar shapes. Skewness and kurtosis describe the asymmetry and peakedness of a distribution, respectively. The skewness is the third standardized moment of a distribution. For a sample of size *n*, it is defined as *skew *= . The skewness is positive for distribution with a tail to the right, and negative for left-tailed distributions as illustrated in Figure 3. The kurtosis is defined as *kurtosis *= . It measures how sharply peaked a distribution is, relative to its width. We subtract 3 to achieve a kurtosis of zero for the Gaussian distribution. A distribution with positive kurtosis has more probability mass around the mean than the Gaussian distribution whereas a distribution with negative kurtosis has less probability mass around the mean and is therefore less peak-shaped. We give an example in Figure 4.

• Minimum and maximum intensity of the TIC. We store the maximum and minimum intensity over the whole LC-MS run.

Using the descriptors described above, we can now represent an LC-MS map as a vector  where each entry of this vector represents one of the quality descriptor described above.

#### Outlier Detection using the Mahalanobis Distance

To decide whether an LC-MS map is an outlier compared to the rest of the measurements, we use the Mahalanobis distance [23]. It has previously been applied in numerous tasks, such as the quality assessment of microarray experiments [20] or face recognition [24]. It is related to the Euclidean distance but differs in the fact that each dimension is weighted by its variation.

Formally, the Mahalanobis distance of a vector  = (*x*_1_, *x*_2_,...*x*_*p*_) to a distribution with mean  = (*μ*_1_, *μ*_2_,...*μ*_*p*_)^*T *^and covariance matrix Σ ∈ *R*^*p *× *p *^is defined as:

(3)

Using the Mahalanobis distance, we can therefore measure the distance of each LC-MS run, described by the vector of its quality descriptors , to the distribution of all other *n *runs, characterized by their mean vector  and covariance matrix Σ. The Mahalanobis distance of a vector with dimension *p *follows a *χ*^2 ^distribution with *p *degrees of freedom. This allows us to define cutoffs for suspiciously large distances for a given confidence level *α *as in any statistical test. Note that the Mahalanobis distance is equal to the Euclidean distance if the covariance matrix is the identity matrix. In this case, each dimension has unit variance and each pair of dimensions is uncorrelated.

Note that if we use this criterion for outlier detection, we effectively classify a map as outlier if its vector of quality descriptors differ by a large extent from the rest. This is reasonable since even for non-replicate LC-MS runs, we would expect most quality descriptors to be similar.

However, this approach suffers from two drawbacks: first, for less LC-MS runs than descriptors (*n *<*p*), the covariance matrix Σ is singular and cannot be inverted. Second, outlier in the data might distort our estimates of  and Σ and lead to incorrect estimates of the distance. We solve the first problem by applying a Principal Component Analysis to reduce the dimensionality of our data to a dimension *p' *≪ *p *but try retain the essential information at the same time. We solve the second problem by using robust estimators for location and scale.

#### Robust Principal Component Analysis

Principal Component Analysis (PCA) [25] is a method for dimensionality reduction and feature extraction. More formally, our aim is to represent a vector  by a lower dimensional representation given by *M* =  where *M *is a matrix with dimension *dim*(*y*) × *dim*(*x*) where *dim*(*y*) <*dim*(*x*). *M *represents a projection from the higher dimensional space of  to a lower dimensional space of . In the case of PCA, *M *is an orthogonal linear projection.

The standard PCA works as follows: the data, in our case the vectors of quality descriptors for each map, are stored in a *n *× *p *matrix *X *with a row for each of the *n *maps and a column for each of the *p *quality descriptors. This matrix is centered by subtracting the column-wise mean. The covariance matrix Σ of the data is given by *X*^*T *^*X*. It contains the variance of each dimension *p *on its diagonal and the covariances in the remaining entries. We compute the eigenvectors of the covariance matrix and choose as coordinates the eigenvectors with the largest eigenvalues. We project the data into a lower dimensional space by computing  = *E* where *E *contains the chosen eigenvectors as column vectors.

The standard PCA approach is sensitive to outliers. Outlier points will lead to wrong estimates of center and variance and thus distort the results of the projection. To remedy this, we use a robust version of Principal Component Analysis (rPCA). In statistics, an approach is considered robust if it is not, or not severely, influenced by outlier observations. We use a rPCA algorithm developed by Croux *et al*. [26]. First, we center the data using a robust estimator of location, the *L*_1 _median:

(4)

The *L*_1 _median is simply the point *θ*, not necessarily a data point, which minimizes the Euclidean distance to all other points. In contrast to the simple component-wise mean or median for multivariate data, the *L*_1 _median gives a robust estimate of the center and is invariant to orthogonal linear transformations such as PCA. Efficient algorithms for its computation exist [27].

Furthermore, we compute a robust PCA using projection pursuit [26]. In short, the projection pursuit approach to PCA examines a finite subset of all possible directions of the data in the measurement space. In our case, the set of candidate directions is given by the centered observations themselves. We approximate the first direction which maximizes a robust estimator of scale and choose all following direction vectors to be orthogonal to the previous one. We use the Median of Absolute Deviation (MAD) to estimate the scale

(5)

where *c *is a correction factor reflecting our assumption that the data is normally distributed and equals 1.4826. The projection pursuit approach to PCA also gives a robust estimate of the covariance matrix [26] which we use for the computation of the Mahalanobis distance.

After projecting the data into the subspace of the direction with the highest robust variance, projection pursuit searches for an approximation of the next eigenvector which is assumed to be orthogonal to the previous one. Obviously, one needs to decide on the dimensionality of the subspace the data is projected into. We always choose a number of components that would explain 90% of the variance, which is usually around 6. Consequently, we can now describe each LC-MS map using a vector in 6 dimensions instead of 20, the covariance matrix Σ is invertible and we can search for suspicious maps by plotting the Mahalanobis distance for each map. Note that each dimension does not longer represent a unique descriptor, but a linear combination of all descriptors. But by inspecting the weights (also called *loadings *in PCA terms) we can gain insights into which descriptor contributed the most to a particular dimension. To summarize, our method aims at the identification of poor runs among a group of LC-MS experiments. Our method assumes that the majority of the runs are good and that poor runs differ significantly in their quality descriptors from the rest. Consequently, what constitutes a good and a poor run is determined by the majority of the experiments. Since our method is robust, it suffices if at least half of the LC-MS runs are good.

### Implementation

The programs to compute the quality descriptors for an LC-MS map were written using OpenMS [28], our software library for computational mass spectrometry. We performed the statistical analysis and visualization of the results using the mathematical software package R .

### Testing

We present three use cases to demonstrate how our approach can be applied to automatically detect outlier runs among a set of LC-MS maps. We start with a set of simulated maps. The simulation allows us to probe the capabilities of our approach on a detailed level. The second and third use case comprise a tryptic digest of bovine serum albumin (BSA) and urine samples from a healthy volunteer recorded using LC-ESI-MS.

Details of the full mass spectrometry analysis concept and chromatographic setup is described elsewhere [29]. In short, we employed a restricted access sulphonic acid strong cation-exchanger (RAM -SCX) (Merck KGaA, Darmstadt, Germany) column followed by a peptide transfer and solvent switch through trap column (Chromolith Guard, 5 × 4.6 mm Merck KGaA). We performed a subsequent analysis using an analytical column (Chromolith CapRod RP18e, 150 × 0.2 mm, Merck KGaA) by means of column switching to perform two dimensional orthogonal separations. On-line mass spectrometric detection was performed using an Esquire Series 3000 PLUS ESI Iontrap mass spectrometer (Bruker Daltonics, Bremen, Germany).

The BSA digest was prepared according to a standard procedure (Proteo Extract All-in-one Trypsin Digest Kit, Merck Chemicals Ltd, Nottingham, UK) with final concentration of 2 mg/ml and stored at 20°C. Urine samples were from healthy volunteers pooled and stored at 20°C. Before analysis samples were defrosted at room temperature for an hour, and then filtered through 0.22*μ*m pore size low protein binding membrane filters (Durapore, Millipore) and clear sample transferred to autosampler tubes. The prepared samples were stored in an autosampler at 4°C not longer than 24 h before injection.

#### Simulated LC-MS runs

**Figure 5:**
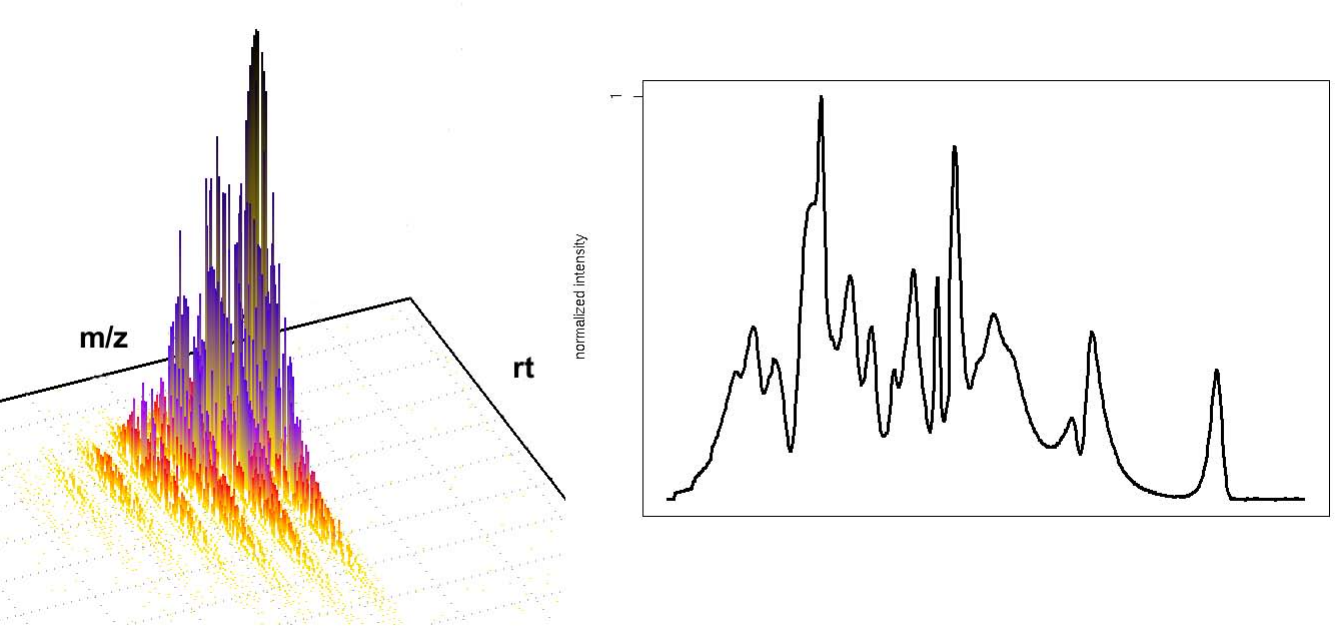
**A simulated LC-MS map**. A simulated LC-MS map (without noise, i.e. not an outlier): 3D view of a peptide ion signal from this map (left) and the TIC (Total Ion Chromatogram) of the map (right).

To provide a sanity check of our method, we simulated a mixture of standard peptides using our software *LC-MSsim *[30]. This software simulates an entire LC-MS experiment, including protein digestion, retention time prediction, isotopic pattern and elution peak models. It produces a realistic LC-MS map and the user can introduce noise, non-peptidic contaminants, m/z and intensity errors at its own will. We simulated a mixture consisting of peptides from a tryptic digest of 4 standard proteins, namely bovine catalase, horse myoglobin, bovine carbonic anhydrase and bovine lysozyme. To test our approach, we simulated 20 perfectly reproducible runs and 10 outlier. We simulated the good LC-MS maps by introducing only limited noise but added a high amount of shot noise to each outlier map. The shot noise was simulated using a Poisson distribution with rate 1000 and an intensity mean of 1600 for outlier maps and for good runs with rate 100 and mean 500. Figure 5 gives an illustration of a simulated LC-MS map with good quality.

**Figure 6:**
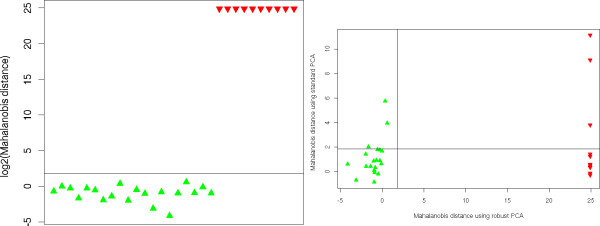
**Sanity check using simulated LC-MS runs**. Left: Log-scaled Mahalanobis distances for the simulated LC-MS runs. The black line gives a cutoff for a significance level of 5% as computed from the *χ*^2 ^distribution. Right: Comparison of Mahalanobis distances computed using standard (y-axis) and robust PCA (x-axis). Good runs are given in green up-pointing triangles and simulated outlier are given in red down-pointing triangles. The vertical and horizontal lines indicate the 5% cutoff for both distances. This plot shows that the robust approaches have advantages if the data contains outlier: using standard PCA we would not classify most of the simulated outlier correctly, furthermore standard PCA classifies several good maps as outlier.

Our aim was investigate whether our approach based on quality descriptors, Mahalanobis distance and robust principal component analysis would be able to recover all outlier runs correctly. Figure 6 (left) shows that the robust Mahalanobis distance highlights all the simulated outlier (red down-pointing triangles) which have a much higher distance than the good maps (green up-pointing triangles). The black line gives indicates the threshold for a 0.05 confidence level and a Bonferroni correction for multiple testing. Furthermore, Figure 6 (right) shows a comparison of the robust Mahalanobis distance versus non-robust version of this distance (i.e. without robust PCA and robust estimator of location). The black lines indicate the cutoff for a confidence level of *α *= 0.05 with the Bonferroni correction for multiple testing. All points above the horizontal line are outlier according to the standard PCA of the quality descriptors. Points to the right of the vertical line represent maps that classified as outlier using a robust PCA, both at a confidence level of 0.05. This plot shows that it actually makes sense to use a robust approach since the standard approach would incorrectly classify several outlier as good data set. Furthermore, an outlier detection computed using a non-robust PCA would also classify several of the good LC-MS maps as outlier. Nevertheless, simulated data can only provide a sanity check of a computational method and thus we will provide an evaluation on real data in the following two sections.

#### Tryptic Digest of Bovine Serum Albumin

This data set are replicate LC-MS recordings of a tryptic digest of bovine serum albumin (BSA). The peptide mixture was measured in 43 replicates, details of sample preparation and LC-MS analytics are described elsewhere [29]. Using the algorithms implemented in TOPP/OpenMS [28,31], we performed peptide feature detection, alignment and statistical analysis for these runs. After manual inspection, we classified 5 runs as outlier for various reasons: 3 exhibited peptide feature intensities that deviated by a large extent from the other replicates. The remaining two revealed significant shifts in retention time as compared to the remaining runs. This fact made an alignment difficult and required manual fine-tuning of the alignment algorithm.

This is of course a time-consuming procedure. It would be preferable to have a method that would allow us to remove outlier before feature detection and alignment is performed to save time and computer resources. Consequently, we applied our quality assessment method to these runs.

**Figure 7:**
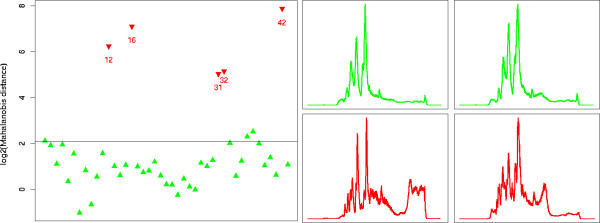
**Results on LC-MS maps of a BSA digest**. Left: Log-scaled Mahalanobis distances for the BSA digest. The black line gives a cutoff for a significance level of 5%. Good runs are given in green up-pointing triangles and simulated outlier are given in red down-pointing triangles. The numbers denote the time order of the runs, but are only given for the outlier runs. Right: Examples of LC-MS maps classified as good (green) and outlier (red).

Figure 7 (left) gives for each of the 43 replicates the Mahalanobis Distance *D*_*M *_to the center of all other measurements. LC-MS maps that were manually identified as outlier are given as red down-pointing triangles, good replicates as green up-pointing triangles. The horizontal line gives a cutoff corresponding to a significance test with *α *= 0.05 and Bonferroni correction for multiple testing. In other terms, all LC-MS maps with distances above this threshold are classified as outlier by our method.

As we can see from Figure 7 (left), the combination of spectral quality descriptors, robust principal component analysis and Mahalanobis distance is accurate and classifies all outlier maps correctly. It also classifies some additional maps as mild outlier, namely the first LC-MS map and the maps with number 36 and 37. Manual inspection of the PCA loadings revealed that their larger Mahalanobis distances are mainly due to a higher noise level in some spectra and minor fluctuations in the TIC. For illustration, Figure 7 (right) shows the TIC of four maps. Again, normal runs in the upper row are colored in green, outlier runs are colored in red. Both outlier maps exhibit TICs that contain a significant amount of noise peaks and clearly deviate from the two good runs in the top row.

#### Urine Samples of a Healthy Volunteer

This data set consists of 54 LC-MS runs. A manual inspection indicated that five of these runs are clear outlier. Four of these five runs were measured after a break of several days which seems to have lead to disturbances in the chromatography and sample composition. The fifth outlier has a significantly elevated noise level.

**Figure 8:**
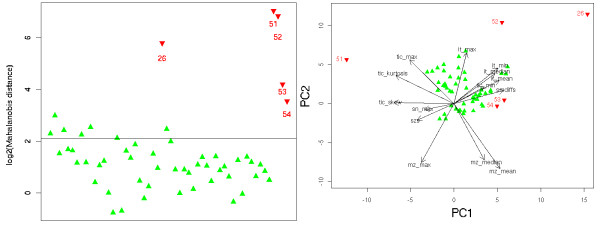
**Results on LC-MS maps of urine samples**. Left: Log-scaled Mahalanobis distances for the renal patient samples. The black line gives a cutoff for a significance level of 5%. Outlier are given red down-pointing triangles and their run numbers. Right: Projection of the LC-MS maps on their first two principal components. This plot shows that the data (scores) projected on the first two principal components and the variables (loadings) plotted as arrows.

Figure 8 (left) gives the Mahalanobis distances for this data set. Runs that were classified as outlier by manual inspection are given as red down-pointing triangles, normal runs as green up-pointing triangles. The numbering of data indicates the time order of runs. As we can see, all known outlier maps are recovered. Additionally, some normal runs are classified as mild outlier. Due to the complex composition of the samples, it is difficult to judge whether these runs comprise true outlier that were not discovered during the manual inspection or not. In a real-world study without enough time to perform a manual validation, one would discard the strong (and true) outlier maps. Depending on time and lab resources available, we would recommend to treat the mild outlier with caution or even to repeat these experiments. Figure 8 (right) shows the projection of the maps onto the first two principal components of their quality descriptor vectors. This plot is called a biplot. Interestingly, we see that the LC-MS maps form two clusters: maps measured before the strong outlier run 52 fall into one cluster, all maps measured after 52 fall into another cluster. This exhibits one potential weakness of the Mahalanobis distance if used for outlier detection: the data is expected to form a single cluster and the method gives less reliable results of this is not the case. This is also the reason why several well reproducible runs were classified as mild outlier. Furthermore, the biplots shows the variables (loadings) as arrows. We can see that outlier 51 stands out because of the skewness, kurtosis and maximum intensity value of its TIC. The remaining outliers differ from the good runs mainly because of their elevated intensity values and higher noise content.

## Discussion

Quality assessment and control are common place in fields where many items are produced at a rapid pace and where quality is crucial: be it tools in factories or data in high-throughput biological experiments. The application of statistical quality assessment to quantitative mass spectrometry data is still an underexplored field. We expect that, with the growth of this field, this is going to change as much as it has changed for gene expression studies.

We presented a statistical method for outlier detection in large scale mass spectrometry studies. It is based on quality descriptors capturing different aspects of the quality of an LC-MS map and on a statistically robust version of the Mahalanobis distance. We demonstrated that our approach works well with large data sets and can accurately detect poor LC-MS runs. This is of special importance in high-throughput experiments, where many LC-MS maps are generated and the time lacks to perform a manual quality assessment.

We evaluated our approach on simulated LC-MS runs and two real data sets consisting of around 50 replicates each. In all cases, we were able to detect outlier data sets, outlier that were confirmed by manual validation. When dealing with outlier, we have two choices: to either remove them or to repeat the corresponding LC-MS run. Clearly, this depends on the time and lab resources available. In each case, outlier detection and removal as early as possible during the data analysis will make the results more reliable and save a lot of time and computational effort.

## Competing interests

The authors declare that they have no competing interests.

## Authors' contributions

OS-T designed the method and drafted the manuscript. EM, HS and JT recorded the LC-MS maps and gave recommendations for their evaluation. KR and KU designed the study and provided feedback and directions on the results. All authors have read and approved the final manuscript.
